# Regional variations in geographic access to inpatient hospices and Place of death: A Population-based study in England, UK

**DOI:** 10.1371/journal.pone.0231666

**Published:** 2020-04-17

**Authors:** Emeka Chukwusa, Peihan Yu, Julia Verne, Ros Taylor, Irene J. Higginson, Gao Wei

**Affiliations:** 1 Department of Palliative Care, Policy and Rehabilitation, King’s College London, Cicely Saunders Institute, London, United Kingdom; 2 Knowledge & Intelligence (South West), National End of Life Care Intelligence Network, Public Health England, Bristol, United Kingdom; 3 Royal Marsden NHS Hospital Trust, London, United Kingdom; 4 Hospice UK, London, United Kingdom; University of Oxford, UNITED KINGDOM

## Abstract

**Background:**

There is much variation in hospice use with respect to geographic factors such as area-based deprivation, location of patient’s residence and proximity to services location. However, little is known about how the association between geographic access to inpatient hospice and hospice deaths varies by patients’ region of settlement.

**Study aim:**

To examine regional differences in the association between geographic access to inpatient hospice and hospice deaths.

**Methods:**

A regional population-based observational study in England, UK. Records of patients aged ≥ 25 years (n = 123088) who died from non-accidental causes in 2014, were extracted from the Office for National Statistics (ONS) death registry. Our cohort comprised of patients who died at home and in inpatient hospice. Decedents were allocated to each of the nine government office regions of England (London, East Midlands, West Midlands, East, Yorkshire and The Humber, South West, South East, North West and North East) through record linkage with their postcode of usual residence. We defined geographic access as a measure of drive times from patients’ residential location to the nearest inpatient hospice. A modified Poisson regression estimated the association between geographic access to hospice, comparing hospice deaths (1) versus home deaths (0). We developed nine regional specific models and adjusted for regional differences in patient’s clinical & socio-demographic characteristics. The strength of the association was estimated with adjusted Proportional Ratios (aPRs).

**Findings:**

The percentage of deaths varied across regions (home: 86.7% in the North East to 73.0% in the South East; hospice: 13.3% in the North East to 27.0% in the South East). We found wide differences in geographic access to inpatient hospices across regions. Median drive times to hospice varied from 4.6 minutes in London to 25.9 minutes in the North East. We found a dose-response association in the East: (aPRs: 0.22–0.78); East Midlands: (aPRs: 0.33–0.63); North East (aPRs: 0.19–0.87); North West (aPRs: 0.69–0.88); South West (aPRs: 0.56–0.89) and West Midlands (aPRs: 0.28–0.92) indicating that decedents who lived further away from hospices locations (≥ 10 minutes) were less likely to die in a hospice.

**Conclusion:**

The clear dose-response associations in six regions underscore the importance of regional specific initiatives to improve and optimise access to hospices. Commissioners and policymakers need to do more to ensure that home death is not due to limited geographic access to inpatient hospice care.

## Background

The increased prevalence of chronic illnesses in an ageing population has led to an increasing proportion of people needing palliative care globally [1] [2, 3]. In 2014, the World Health Organisation Assembly called to incorporate palliative care into the healthcare systems of member countries [4]. However, currently only an estimated 14% of people who require such care do receive it [5], even though access to high-quality palliative care is considered a human right [6, 7] [8]. Improving access to palliative care depends on a thorough understanding of factors associated with utilisation of palliative care services and how they vary geographically.

In the UK, the growth of hospice began with the modern hospice movement in St Christopher’s by Dame Cicely Saunders in 1967 [9]. Since then, hospices have developed spontaneously with donations from charitable and voluntary organisations, which may be a contributory factor to inequalities of hospice provision [10]. Studies in the UK and elsewhere have highlighted variations in the utilisation of hospice with respect to geographic factors such as area-level deprivation, place of residence/settlement types, drive time or distance from hospice locations. For example, there is evidence that those who live in deprived neighbourhoods are less likely to die in a hospice[11, 12] or receive hospice at home care [13]. A study by Gatrell and Wood showed that deprived areas have poor geographic access to hospices [14]. Recent evidence from a systematic review and meta-analysis involving studies from high-income countries has also shown that there are inverse associations between area deprivation and receipt of specialist palliative care, including hospice use [15]. In addition to area-level deprivation, several US-based studies found subnational variations in the use of and duration of hospice enrolment [16] [17] including frequency of hospice visits by professionals in the last 2-days of life [18].

Geographic variations in hospice use/deaths may be partly due to within-country differences in geographic access to and/or availability of hospices. To date, there is little understanding of subnational variations in the association between geographic access to hospice and hospice use. Existing studies have used national level estimates, which can mask nuances in regional level geographic access. For example, in a previous national population-based study we found rural-urban variations in geographic access to hospices, with urban dwellers having better access to inpatient hospice facilities compared to their rural counterparts [19]. Our findings were, however, limited in the extent to which they may be generalisable to regions within England, UK. One UK study using cancer data for only one region (North West), found that patients are more likely to die in a hospice if they lived closer to the facility [20].

Understanding regional level variations in the association between geographic access to hospice and hospice use and/or hospice deaths is essential to identify where there may be gaps or inequities in service provision. For example, inverse associations between geographic access to hospice and hospice death in some regions may indicate inequality or barriers to hospice provision. Knowledge of regional variations is also useful to help guide care planning, develop policies for targeted service improvement and allocate resources to regional needs more appropriately. We aimed to examine regional variations in geographic access to adult inpatient hospice and to explore regional differences in the association between geographic access to hospice and hospice deaths (comparing hospice *vs* home deaths) in England, UK. This research is timely, given that the improvement of access to palliative care is a priority of the World Health Organisation Assembly [4].

## Methods

### Study design and settings

A regional population-based observational study in England, UK. The study followed the STROBE Statement for reporting of observational studies using routinely collected health data [21]. Regions were defined using the 2011, Government Office Region (GOR) classification. There are nine GORs in England (London, East Midlands, West Midlands, East Yorkshire and the Humber, South West, South East, North West and North East). The National Health Service (NHS) in England has regional teams, working closely with General practices, health wellbeing boards, local authorities and Clinical Commission Groups (CCGs) to commission and deliver care for their local population [22]. CCGs are planning regions for commissioning health care in England, UK.

### Data sources and study population

All non-accidental (i.e. excluding deaths from external causes such as accident, suicide or poison) adult deaths (aged ≥ 25 years), consisting of those who died in an inpatient hospice facility or at home in the year 2014, were extracted from the ONS death registry. We used 2014 data as this was the most recent year of cleaned and checked data at the time of analysis.

The data includes the age of the deceased, gender, causes of death (CoD), marital status, number of contributory causes of death (NCoD), which are the causal sequence of events leading to death [23]. The CoD and NCoDs were recorded in the death registry according to the International Classification of Diseases 10th edition (ICD-10). Deaths were allocated to each of the nine GORs in England (London, East Midlands, West Midlands, East, Yorkshire and The Humber, South West, South East, North West and North East) through record linkage with postcode of usual residence of the deceased. Death records were linked to area-level index of multiple deprivation and settlement types (rural-urban indicator) based on the residential postcode of the deceased. The index of multiple deprivation is an area-based estimate of socioeconomic status (SES) for each Lower Super Output Area (LSOA) level grouped into quintiles (1: Most deprived and 5: Least deprived). LSOAs are geographic units with an average population of 1500 persons [24].

**Fig 1:**
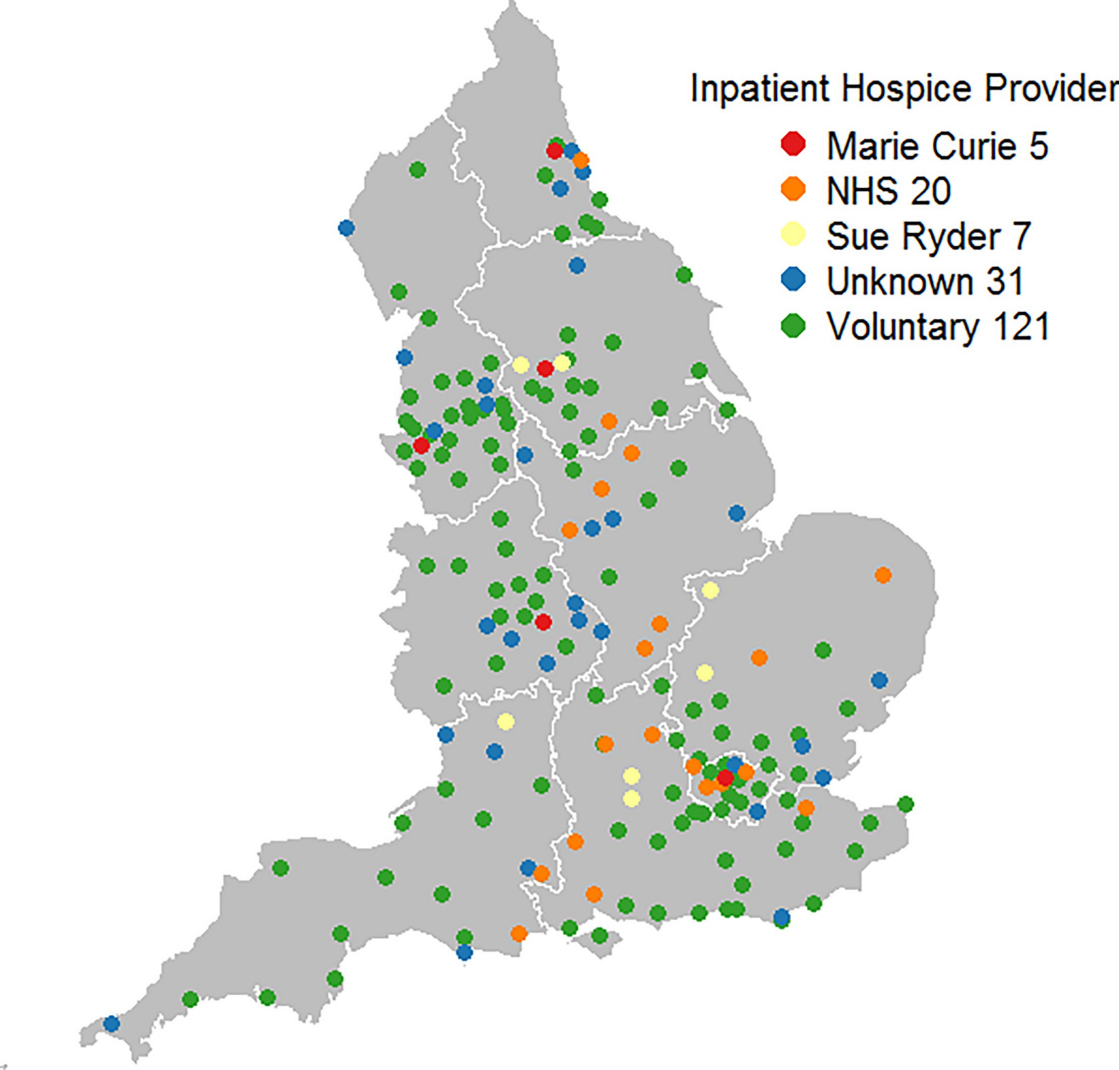
Geographical distribution of Inpatient hospices by providers. The digital boundary file contains Office for National statistics data © Crown copyright and database right (2016) and contains Ordnance Survey data © Crown copyright and database (2016) Source of hospice data: https://hospiceaid.org.uk/.

Hospice data were downloaded from the Hospice aid UK website [25]. It comprised of adult inpatient hospices (n = 184) (including hospices managed by the NHS, charities or voluntary organisations) in England (Fig 1). We focussed on hospices providing adult services because our cohort comprised of adults (i.e. patients aged 25 years and above).

Road network data used for calculating drive times to hospice, were downloaded from the University of Edinburgh data library [26]. The original data was in a geographic mark-up language format but was converted to shapefiles to enable calculation of drive times. All shapefiles used for geographical mappings were downloaded from the UK data services (https://www.ukdataservice.ac.uk/get-data). Data were checked and cleaned for errors.

### Ethical approval and permission

Ethical approval was granted by the King’s College London Research Ethics Committee for secondary analysis of death registration data (reference number: BDM/14/15-5). The study used fully anonymised datasets and did not involve direct patient contact. Hence, consent to participate was not required. Data access agreement was signed, and all required forms provided in a formal agreement of data management, protection, and management. In addition, as required, all researchers accessing the data (EC, WG, and PY) were individually assessed and approved by the ONS.

### Variable definitions

The dependent variable was binary (1 = Hospice versus Home = 0). Geographic access was the explanatory variable analysed in categories (0–10 minutes, 10–30 minutes, 30–50 minutes and Over 50 minutes). We analysed access as categorical variable to facilitate easy comparison across regions and to compare findings with previous study [19]. Geographic access was measured by calculating drive times from patients’ place of residence to nearest hospices taking into account the speed limits of various road types. Drive times were calculated separately for each region. We did not consider variations in traffic conditions in our drive time calculation, due to lack of traffic data. Calculation of drive times were completed in ESRI ArcGIS using the Network Analyst extension [27].

Confounding variables included patients’ age at death (25–54, 55–64, 65–74, 75–84 and 85+), gender (male & female), marital status (divorced, married, separated/dissolved, single, unknown/not stated and widowed), NCoDs (0, 1, 2, 3, 4, and 5+), type of settlement (rural and urban), SES and CoD. CoD was grouped into six categories: Cancers (ICD-10: C00 –C97), Neurological conditions (ICD-10: G35—G37, G20, F02.3, G12), Chronic Obstructive Pulmonary Diseases COPD (ICD-10: J40—J44, J47), CVDs (ICD-10: I00 -I52, I70—I99), Cerebrovascular diseases (CBDs: G45 –G46, I60 –I69), and other non-accidental cause of deaths.

### Statistical analysis

Patients’ socio-demographic and clinical characteristics were described using percentages. Variations in geographic access to hospice across regions were described using median and interquartile range (IQR) of drive time values. CCG-level median drive time values nested within regions, were mapped and variations in drive time were displayed using Choropleth maps.

A Modified Poisson regression [28] was used to estimate the association between geographic access to hospices, comparing hospice deaths (1) and deaths at home (0). We developed nine regional-specific models and adjusted for patients’ socio-demographic and clinical characteristics shown in Table 1.

**Table 1:** Drive time and patients socio-demographic characteristics by regions.

Variables	Values	East	East-Midlands	London	North-East	North-West	South-East	South-West	West-Midlands	Yorkshire and the Humber
N	N = 123088	14324	10589	12386	6797	17434	20618	13922	13358	13660
%		11.63	8.60	10.0	5.52	14.2	16.8	11.3	10.9	11.1
Drive time (Minutes)	Median Distance to hospice	10.1	16.9	4.6	25.9	10.1	9.4	15.8	6.7	24.8
	Interquartile range (IQR)	6.0–19.2	8.5–29.1	3.3–6.1	18.5–33.8	6.1–20.6	5.6–15	8.8–31.9	4.8–9.4	15.5–34.2
Age group	25–54	7.5	7.6	9.5	8.2	8.5	7.3	6.9	8.1	8.4
	55–64	11.2	11.7	12.8	14.9	13.5	11.5	11.4	12.8	13.0
	65–74	22.7	24.8	21.4	25.3	25.6	22.6	22.6	23.8	25.1
	75–84	31.0	31.1	29.0	30.5	31.2	31.1	30.3	31.0	31.4
	85+	27.7	24.7	27.3	21.2	21.1	27.5	28.8	24.2	22.2
Gender	Female	46.8	46.4	47.8	46.2	46.8	47.0	45.6	46.2	45.9
Marital Status	Male	53.2	53.6	52.2	53.8	53.2	53.0	54.4	53.8	54.1
	Divorced	10.9	10.9	12.2	11.8	12.6	11.1	10.9	10.8	11.9
	Married	50.8	51.1	40.5	47.3	47.3	50.5	51.8	49.5	49.5
	Separated/Dissolved	0.2	0.1	0.3	0.1	0.1	0.2	0.2	0.1	0.1
	Single	7.3	7.3	16.1	9.7	9.6	8.2	7.6	8.8	9.0
	Unknown/Not Stated	0.5	0.4	1.4	0.4	0.4	0.4	0.5	0.4	0.4
	Widowed	30.3	30.2	29.6	30.8	30.0	29.6	29.1	30.3	29.1
Underlying cause of death (CoD)	Cancers	35.4	36.3	34.8	37.8	39.0	36.4	35.9	36.6	36.8
	CBDs*	5.9	5.3	5.4	6.2	5.2	5.7	6.1	5.7	5.8
	COPDs*	4.3	5.0	4.6	4.6	4.6	4.6	5.1	4.6	4.2
	CVDs*	23.3	23.9	23.6	22.8	21.9	23.4	23.1	23.2	23.7
	Neurological conditions	1.5	1.4	1.7	1.5	1.5	1.5	1.4	1.6	1.4
	Other Non-accidental Causes of Deaths	29.6	28.0	30.0	27.2	27.8	28.4	28.3	28.3	28.0
Number of Contributory Causes of deaths (NCoDs)	0	36.2	37.0	34.5	38.2	37.3	40.4	39.1	35.6	38.8
	1	27.9	29.7	27.3	28.7	30.8	29.7	30.4	28.7	30.3
	2	18.5	18.0	19.9	18.3	18.4	16.7	17.4	18.8	17.2
	3	10.3	9.2	11.0	9.0	8.3	8.3	8.1	9.9	8.5
	4	4.6	4.0	5.0	4.2	3.3	3.2	3.4	4.6	3.6
	5+	2.5	2.1	2.4	1.6	1.8	1.7	1.7	2.4	1.6
Socioeconomic Status (SES)	1 (Most Deprived)	9.9	17.1	22.1	33.0	33.6	8.0	10.7	26.5	28.1
	2	18.5	20.3	26.6	24.0	18.1	14.8	19.2	18.0	18.2
	3	25.3	18.9	20.4	15.7	16.8	19.3	25.2	21.1	18.6
	4	21.5	22.8	16.7	13.5	16.7	24.7	24.6	18.7	19.2
	5 (Least Deprived)	24.8	20.9	14.2	13.8	14.8	33.2	20.2	15.6	16.0
Settlement	Rural	33.1	29.5	0.4	19.4	11.6	23.4	35.9	17.8	19.3
	Urban	66.9	70.5	99.6	80.6	88.4	76.6	64.1	82.2	80.7
Place of death	Homes	82.4	84.5	74.7	86.7	78.3	73.0	81.6	78.9	76.7
	Hospices	17.6	15.5	25.3	13.3	21.7	27.0	18.4	21.1	23.3
Number of adult inpatient hospices	Counts	18	13	16	11	30	34	21	22	19

*CBD—Cerebrovascular diseases; COPD—Chronic obstructive pulmonary diseases; CVD—Cardiovascular diseases

The strength of the association was described with adjusted proportional ratios (aPRs). Bonferroni adjustment was used to correct for family-wise error rate. The log-likelihood ratio test was used to assess model fitness. Overall, the models had a good fit at *p* < 0.001. All statistical analyses and GIS maps were completed in R 3.1.3 statistical software [29] using functions from the Maptools [30] and GISTool libraries [31].

## Results

### Patients’ clinical and socio-demographic characteristics across regions

There were 123088 recorded home and hospice deaths in England in 2014. More than 70% of decedents died at home in all regions (ranging from 73% in the South East to 86.7% in the North East), compared to deaths in inpatient hospice (ranging between 13.3% in the North East to 27.0% in the South East). Cancer was the leading cause of death across all regions. Majority of decedents lived in urban areas (ranging from 99.6% in London to 64.1% in the South West).

### Variations in geographic access to adult inpatient hospice

There were considerable regional variations in individual-level median drive time to hospice, varying from a low drive time value of 4.6 minutes in London to a high of 25.9 minutes in the North East (Table 1). Drive times were less variable in London (IQR: 3.3–6.1); South East: (IQR: 5.6–15.0) and West Midlands (IQR: 4.8–9.4) regions. There was greater variability of drive times in the South West (IQR: 8.8–31.9); North East (IQR: 18.5–33.8), North West (IQR: 6.1–20.6) and Yorkshire & the Humber (IQR: 5.5–34. 5) (Table 1).

**Fig 2:**
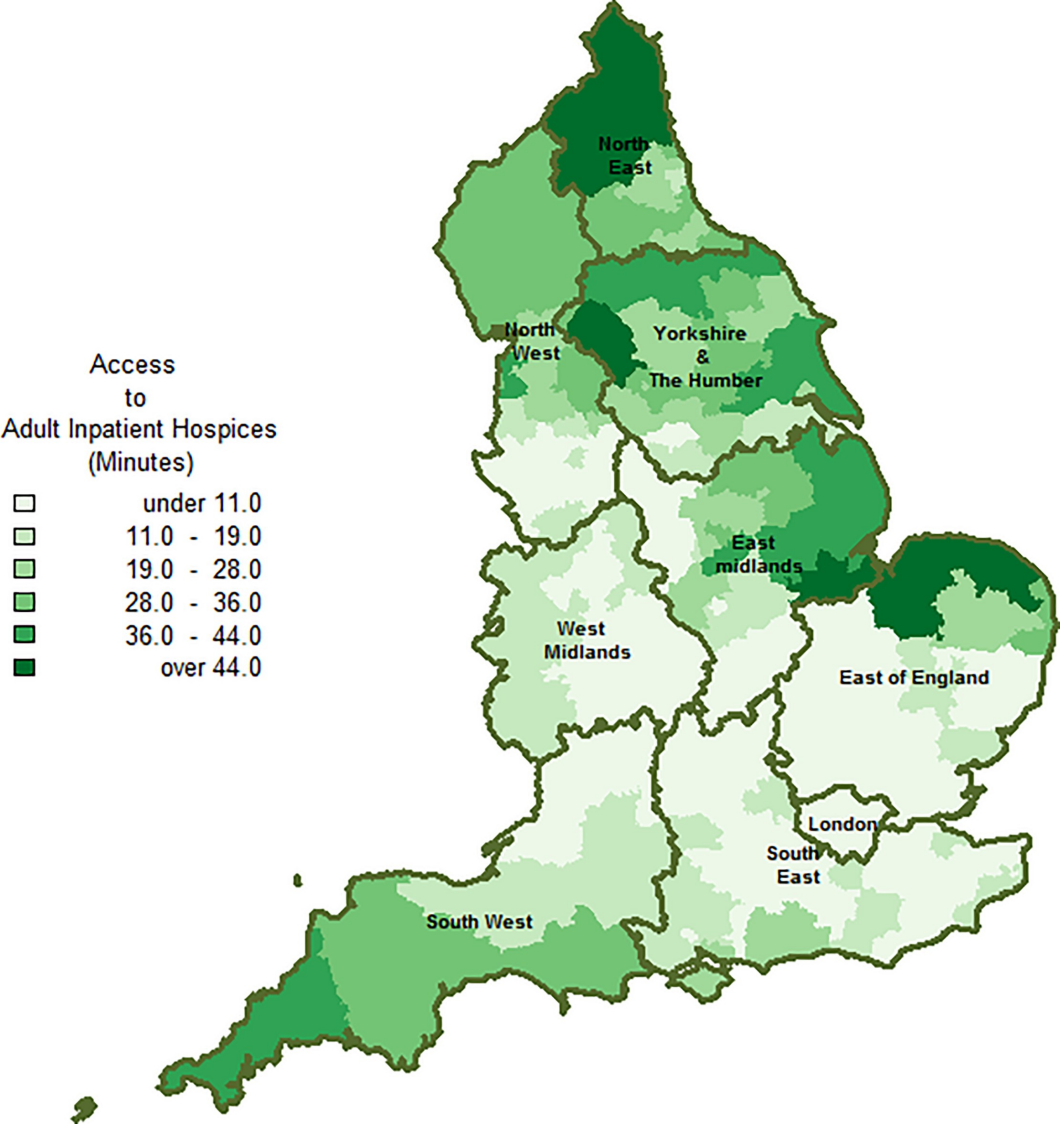
Geographic access to adult inpatient hospice Unit. Drive time from patients’ place of residence to nearest adult inpatient hospice was used as a proxy measure for geographic access. Drive time was computed separately for each region The digital boundary file contains Office for National statistics data © Crown copyright and database right (2016) and contains Ordnance Survey data © Crown copyright and database (2016).

In terms of geographic variation (Fig 2), there was an overall north-south trend in drive times. Drive times were greater (Over 19 minutes) for majority of CCGs in the South West and Northern regions (i.e. North East, North West and Yorkshire and the Humber), compared with CCGs in Central England (i.e. London, West Midlands and parts of East of England).

### Regional variations in the association between geographic access to hospice and hospice deaths

**Fig 3:**
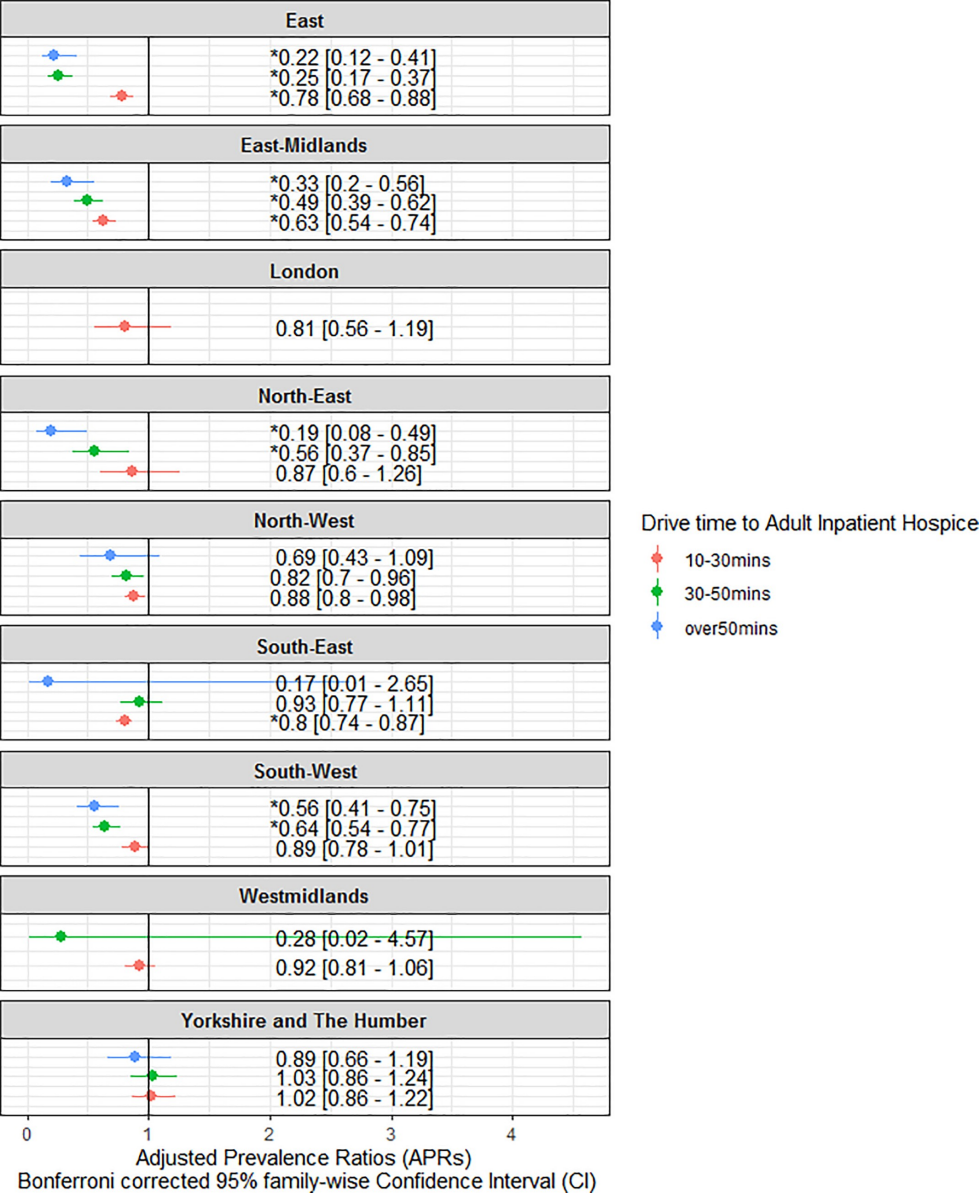
Association between geographic access to adult inpatient hospice and hospice death in nine regions of England. (Hospices Vs Home). Adjusted Proportional Ratios (aPRs) with Bonferroni Corrected 95% family-wise confidence Interval (CI) were derived from modified Poisson regression. aPRs > 1 indicates higher probability of hospice death than the reference category. P-values were adjusted using Bonferroni correction. An asterisks (*) denotes P-value less than or equal 0.005555556 (0.05/9).

Results from modified Poisson regression (Fig 3 and S1 Table) show wide regional variations in the likelihood of hospice deaths across drive time categories 10 to 30 minutes (aPRs: 0.63–1.02); 30 to 50 minutes: (aPRs: 0.24–1.03); and Over 50 minutes: (aPRs 0.16–0.88). There were decreased chances of hospice deaths across all regions, except in Yorkshire and the Humber, where patients who lived within 10 to 30 minutes (aPR: 1.02: 95% CI. 0.93–1.13, p = 0.64) and 30 to 50 minutes’ drive times (aPR: 1.03, 95% CI 0.93–1.15), had marginal chances of dying in a hospice. Patients who lived furthest (i.e. ≥ 50 minutes’ drive time) from hospice locations in the East (aPR: 0.22, 95% CI. 0.15–0.33); East Midlands (aPR: 0.33, 95% CI. 0.23–0.47); North East (aPR: 0.19, 95% CI. 0.10–0.36); North West (aPR: 0.69, 95% CI. 0.51–0.93); South East (aPR: 0.17, 95% CI. 0.02–1.18); South West (aPR: 0.56, 95%CI. 0.46–0.67) and Yorkshire and the Humber (aPR 0.89, 95%CI. 0.74–1.06) had the lowest chances of hospice deaths, compared to patients who lived closer (≤ 10 minutes).

There were clear dose-response associations in the East (aPRs: 0.22–0.78); East Midland (aPRs: 0.33–0.63); North East (aPRs: 0.19–0.87); North West (aPRs: 0.88–0.69) and South West regions (aPRs: 0.89–0.56). The dose-response association was only visible for patients who lived within 10 and 50 minutes in West Midlands (aPRs: 0.28–0.92) because the entire cohort lived within a 50 minutes drive time range from nearest hospice locations.

## Discussion

This regional population-based study has shown regional differences in the association between geographic access to hospices and the probability of dying in a hospice. We found dose-response associations in six regions (East, East Midlands, North East, North West and South West and West Midlands), indicating that the likelihood of a hospice death reduces with increasing drive times from hospice locations. Our finding is consistent with previous studies [20] which showed that greater distance or drive times from a healthcare facility reduced the probability of dying in the facility. In addition, the observed regional differences in the likelihood of hospice deaths, demonstrates the importance of exploring regional level variations in geographic access, which can be obscured when analyses are conducted at a national level.

Our findings of dose-response associations suggest that greater drive times from hospice locations may be a limiting factor for hospice deaths in the East, East Midlands, North East, North West and South West and West Midlands. However, caution must be exercised in interpreting this finding as our model did not account for regional differences in the number of hospice beds or community outreach teams. Furthermore, we had not consider patients’ preferences in place of death in our analysis, which might have confounded our findings because hospice and home are both preferable places of death [32]. We did not find a dose-response association in Yorkshire and the Humber, London, and the South East, suggesting that other factors might be more important than drive times to hospice alone. The Yorkshire and the Humber region had a distinct pattern of association from the rest regions. Patients who lived less than 50-minutes’ drive time from hospice locations had a marginal likelihood of dying in a hospice. This distinct pattern may be due to local preferences for dying in an inpatient hospice or perhaps those living closer to a hospice are already receiving hospice care in their own home.

We found that patients who lived furthest (i.e. ≥ 50 minutes’ drive time) from hospice locations in all regions, had the lowest chance of dying in a hospice and are therefore more likely to die at home. Given the consistency of this finding across regions, it is reasonable to conclude that the high likelihood of home death may be due to limited geographic access to inpatient hospice care or perhaps due to lack of other alternative places of care (e.g. hospital). For example, a previous study using data from North West England showed that living further from a hospice increases the likelihood of hospital death [20]. Other factors not considered in our model such as policy, may have contributed to the high likelihood of home death. The UK national end of life strategy [33], which was rolled out in 2008, to encourage and promote home deaths may also explain why home deaths are more likely in most regions. Preferences for home death may also be a contributory factor. A previous population-based survey found that between 60% to 65% of people prefer to die at home in all regions in England, UK [32].

We found considerable variations in geographic access to inpatient hospice with a north-south trend (Fig 2). The pattern of variations is likely to be multifactorial and may be linked to regional differences in the number of hospice beds or the level of urbanisation in each region. Previous studies have shown that drive times are shorter in urban areas [14]. This may partially explain why drive times are shorter and less variable in highly urbanised regions like London. We did not incorporate variations/intensity of traffic in our drive time calculation. This may have underestimated drive time values in traffic prone regions, such as London. Prospective studies should further explore and understand the mechanisms behind the north-south pattern of geographic variations in drive times by using traffic data.

Our finding of regional variations in the association between geographic access to hospice and hospice deaths has implications for service improvement intervention policies. Policies aimed at improving and enabling people to die in their place of preference should consider regional differences in geographic access to hospices, especially in the six regions, where there are clear dose-response associations. Commissioners and policy-makers need to do more to ensure that home death is not determined by limited geographic access to inpatient hospice care or lack of an alternative place of death. Policy-makers and commissioners should focus on patients' clinical and demographic characteristics in regions where a dose-response association is absent.

### Strength and limitation of study

To our knowledge, this is the first study to explore regional level variations in the association between geographic access to hospice and hospice death in England, UK. Our findings highlight the importance of exploring geographic access at subnational levels. The main strength of the study involves the use of individual-level data. The use of individual-level data minimises the issue of modifiable area unit problem [34], an inherent problem in ecological studies. We estimated the association between access and place of death using PRs rather than Odd Ratios (ORs). The latter has been shown to exaggerate effect size when the outcome of interest is common [28]. Another important strength of this study relates to the large number of influential covariates that we controlled for in our model (Table 1). Nevertheless, our model did not account for the number of community-based services in each region. It is possible that regions with limited geographic access already have well established network of community-based palliative care services. Our model also did not include data on patients’ preference and number of hospice beds. We lacked data to examine the effect of any of these influential factors.

We used hospice data from the hospice aid UK website. This data is by no means comprehensive, some hospices may have been missed. Furthermore, we calculated drive time from patients’ residence to the nearest hospice for each region separately. This stratified approach of calculating drive time does not consider cross-regional travel to hospices. Drive time was used as a surrogate to estimate geographic access, although a superior measure of access but limited by the assumption that patients access hospice with private transport. In addition, drive time calculation did not consider variations in traffic conditions during peak and off-peak times of the day. Finally, we calculated drive time using 2014 death data (6 years old), therefore findings from our analyses may not reflect current situation. More work is needed to explore our regional variations in geographic access to inpatient hospice using current data.

## Conclusion

Our analysis indicates that greater drive times from hospice locations reduced the chances of hospice deaths in six of the nine regions in England, UK, with a dose-response association. This is an indication that geographic access is a limiting factor for hospice deaths. This knowledge could feed into end-of-life service improvement policies of other developed countries with similar health care systems as the UK. For instance, access to inpatient hospice facilities can be improved for patients who want to die in hospices, especially in regions where there are clear dose-response associations. Policy-makers must ensure that patient’s preferences to die at home is not due to lack of geographic access to inpatient hospice facilities.

## Supporting information

S1 Table Association between geographic access and place of death (Home [0] Vs Hospice [1]) for all regions in England 2014. Proportional Ratios (PRs) were estimated from Modified Poisson regression. PR > than 1 indicates a higher likelihood of death at hospice compared to the reference category. PR < 1 suggest lower likelihood of hospice compared to the reference category. Adjusted PRs were derived by adjusting for Age, Cause of Death (COD), Gender, Marital status, Socioeconomic Status (SES) and Number of Contributory Cause of Deaths (NCODs). [Ref–Reference group]. Confidence intervals and p-values were Bonferroni adjusted. An asterisks (*) denotes a P-value less than or equal 0.005555556 (0.05/9).
(DOCX)
