# Supplementary material for: Regional variations in geographic access to inpatient hospices and Place of death: A Population-based study in England, UK
Source: PLoS One. 2020 Apr 17;15(4):e0231666. doi: 10.1371/journal.pone.0231666 (PMC7164606; doi:10.1371/journal.pone.0231666)
Supplement: S1 Table — Proportional Ratios (PRs) were estimated from Modified Poisson regression. PR > than 1 indicates a higher likelihood of death at hospice compared to the reference category. PR < 1 suggest lower likelihood of hospice compared to the reference category. Adjusted PRs were derived by adjusting for Age, Cause of Death (COD), Gender, Marital status, Socioeconomic Status (SES) and Number of Contributory Cause of Deaths (NCODs). [Ref–Reference group]. Confidence intervals and p-values were Bonferroni adjusted. An asterisks (*) denotes a P-value less than or equal 0.005555556 (0.05/9). (DOCX) [file pone.0231666.s001.docx]

**S1 Table:**  Association between geographic access and place of death (Home [0] Vs Hospice [1]) for all regions in England 2014.

| Regions | Geographic  Access  (Drive time) | Unadjusted PR | P -values | Adjusted PR | P - values |
| --- | --- | --- | --- | --- | --- |
| East | 0 – 10mins | Ref |  | Ref |  |
|  | 10-30mins | *0.76 [0.68 - 0.85 ] | *P < 0.001* | *0.78 [0.68 - 0.88] | *P* < 0.001 |
|  | 30-50mins | *0.22 [0.15 - 0.33 ] | *P < 0.001* | *0.25 [0.17 - 0.37] | *P* < 0.001 |
|  | over50mins | *0.18 [0.1 - 0.33 ] | *P < 0.001* | *0.22 [0.12 - 0.41] | *P* < 0.001 |
| East Midlands | 0 – 10 mins | Ref |  | Ref |  |
|  | 10-30mins | *0.64 [0.55 - 0.74 ] | *P < 0.001* | *0.63 [0.54 - 0.74] | *P* < 0.001 |
|  | 30-50mins | *0.47 [0.38 - 0.59 ] | *P < 0.001* | *0.49 [0.39 - 0.62] | *P* < 0.001 |
|  | over50mins | *0.31 [0.18 - 0.52 ] | *P < 0.001* | *0.33 [0.2 - 0.56] | *P* < 0.001 |
| London | 0 – 10 mins | Ref |  | Ref |  |
|  | 10-30mins | 0.88 [0.6 - 1.28 ] | *P = 0.66* | 0.81 [0.56 - 1.19] | *P = 1* |
|  | 30 – 50mins | - |  | - |  |
|  | Over 50mins | - |  | - |  |
| North East | 10-30mins | 0.84 [0.58 - 1.22 ] | *P = 0.77* | 0.87 [0.6 - 1.26] | *P = 1* |
|  | 30-50mins | *0.58 [0.39 - 0.87 ] | *P < 0.001* | 0.56 [0.37 - 0.85] | *P < 0.001* |
|  | Over 50mins | *0.2 [0.08 - 0.5 ] | *P < 0.001* | *0.19 [0.08 - 0.49] | *P < 0.001* |
| North West | 0 – 10 mins | Ref |  | Ref |  |
|  | 10-30mins | 0.89 [0.81 - 0.99 ] | *P = 0.006* | 0.88 [0.8 - 0.98] | *P =0.02* |
|  | 30-50mins | *0.8 [0.68 - 0.93 ] | *P < 0.001* | 0.82 [0.7 - 0.96] | *P =0.02* |
|  | Over 50mins | 0.61 [0.39 - 0.97 ] | *P = 0.011* | 0.69 [0.43 - 1.09] | *P = 0.73* |
| South East | 0 – 10 mins | Ref |  | Ref |  |
|  | 10-30mins | *0.79 [0.73 - 0.85 ] | *P < 0.001* | *0.8 [0.74 - 0.87] | *P < 0.001* |
|  | 30-50mins | 0.95 [0.79 - 1.14 ] | *P = 1* | 0.93 [0.77 - 1.11] | *P = 1.00* |
|  | Over 50mins | 0.14 [0.01 - 2.24 ] | *P = 0.19* | 0.17 [0.01 - 2.65] | *P = 1.00* |
| South West | 0 – 10 mins | Ref |  | Ref |  |
|  | 10-30mins | *0.86 [0.76 - 0.97 ] | *P = 0.002* | 0.89 [0.78 - 1.01] | *P = 0.29* |
|  | 30-50mins | *0.6 [0.5 - 0.71 ] | *P < 0.001* | *0.64 [0.54 - 0.77] | *P < 0.001* |
|  | Over 50mins | *0.51 [0.38 - 0.69 ] | *P < 0.001* | *0.56 [0.41 - 0.75] | *P < 0.001* |
| West Midlands | 0 – 10 mins | Ref |  | Ref |  |
|  | 10-30mins | 1.01 [0.89 - 1.15 ] | *P = 1* | 0.92 [0.81 - 1.06] | *P = 1.00* |
|  | 30-50mins | 0.32 [0.02 - 5.08 ] | *P = 0.75* | 0.28 [0.02 - 4.57] | *P = 1.00* |
|  | Over 50mins | - |  | - |  |
| Yorkshire and The Humber | 0 – 10 mins | Ref |  | Ref |  |
|  | 10-30mins | 0.99 [0.84 - 1.18 ] | *P=1.00* | 1.02 [0.86 - 1.22] | *P = 1.00* |
|  | 30-50mins | 1.00 [0.84 - 1.2 ] | *P=1.00* | 1.03 [0.86 - 1.24] | *P = 1.00* |
|  | Over50mins | 0.8 [0.6 - 1.06 ] | *P =1.00* | 0.89 [0.66 - 1.19] | *P = 1.00* |

Proportional Ratios (PRs) were estimated from Modified Poisson regression. PR > than 1 indicates a higher likelihood of death at hospice compared to the reference category. PR < 1 suggest lower likelihood of hospice compared to the reference category. Adjusted PRs were derived by adjusting for Age, Cause of Death (COD), Gender, Marital status, Socioeconomic Status (SES) and Number of Contributory Cause of Deaths (NCODs). [Ref – Reference group]. Confidence intervals and p-values were Bonferroni adjusted. An asterisks (*) denotes a p-value less than or equal 0.005555556 (0.05/9).
